# Epithelium percentage estimation facilitates epithelial quantitative protein measurement in tissue specimens

**DOI:** 10.1186/1559-0275-10-18

**Published:** 2013-12-01

**Authors:** Jing Chen, Shadi Toghi Eshghi, George Steven Bova, Qing Kay Li, Xingde Li, Hui Zhang

**Affiliations:** 1 Department of Pathology, Clinical Chemistry Division, Johns Hopkins University, 1550 Orleans Street, Cancer Research Building II, Room 3M-03, Baltimore, MD 21231, USA; 2 Department of Biomedical Engineering, Johns Hopkins University, Baltimore, MD, USA; 3 Institute of Biomedical Technology, MBPCG, University of Tampere, Tampere, Finland

**Keywords:** Epithelium, Cancer, Stroma, Computer-aided classification

## Abstract

**Background:**

The rapid advancement of high-throughput tools for quantitative measurement of proteins has demonstrated the potential for the identification of proteins associated with cancer. However, the quantitative results on cancer tissue specimens are usually confounded by tissue heterogeneity, e.g. regions with cancer usually have significantly higher epithelium content yet lower stromal content.

**Objective:**

It is therefore necessary to develop a tool to facilitate the interpretation of the results of protein measurements in tissue specimens.

**Methods:**

Epithelial cell adhesion molecule (EpCAM) and cathepsin L (CTSL) are two epithelial proteins whose expressions in normal and tumorous prostate tissues were confirmed by measuring staining intensity with immunohistochemical staining (IHC). The expressions of these proteins were measured by ELISA in protein extracts from OCT embedded frozen prostate tissues. To eliminate the influence of tissue heterogeneity on epithelial protein quantification measured by ELISA, a color-based segmentation method was developed in-house for estimation of epithelium content using H&E histology slides from the same prostate tissues and the estimated epithelium percentage was used to normalize the ELISA results. The epithelium contents of the same slides were also estimated by a pathologist and used to normalize the ELISA results. The computer based results were compared with the pathologist’s reading.

**Results:**

We found that both EpCAM and CTSL levels, measured by ELISA assays itself, were greatly affected by epithelium content in the tissue specimens. Without adjusting for epithelium percentage, both EpCAM and CTSL levels appeared significantly higher in tumor tissues than normal tissues with a p value less than 0.001. However, after normalization by the epithelium percentage, ELISA measurements of both EpCAM and CTSL were in agreement with IHC staining results, showing a significant increase only in EpCAM with no difference in CTSL expression in cancer tissues. These results were obtained with normalization by both the computer estimated and pathologist estimated epithelium percentage.

**Conclusions:**

Our results show that estimation of tissue epithelium percentage using our color-based segmentation method correlates well with pathologists' estimation of tissue epithelium percentages. The epithelium contents estimated by color-based segmentation may be useful in immuno-based analysis or clinical proteomic analysis of tumor proteins. The codes used for epithelium estimation as well as the micrographs with estimated epithelium content are available online.

## Introduction

The rapid advancement of high-throughput tools for measurement of proteins from cancer tissues or body fluids has demonstrated the potential for the identification of proteins associated with diseases in all areas of medicine. Most of these high-throughput tools utilize either mass spectrometry (MS)-microarray-, or immunosorbent assays for quantitative analysis of proteins [1]. With the advantage of quantitative measurement, currently, many protein assays with good sensitivity and specificity have been developed for research and clinical use in serum, urine and other body fluids. However, the analysis of proteins in tissue specimens is limited to the semi-quantitative immunohistochemistry (IHC) assay that are required to obtain the tissue spatial information and cell type-specific staining patterns. The usage of quantitative protein assays such as MS, microarray, or enzyme linked immunosorbent assay (ELISA) on tissue specimens, however, has its limitations. Due to the loss of spatial information, the measurements acquired are usually confounded by tissue heterogeneity. Since tissue specimens contain various types of cells, where the expressions of target proteins differ, protein assay results become hard to interpret and may even be misleading.

With respect to cancer research, assessment of the expression of epithelial proteins is of great interest, since over 90% of the carcinoma is of epithelial origin [2]. Compared to regions with normal tissue, regions with cancer usually have significantly higher epithelium content yet lower stromal content. Depending on tumor density, the epithelium to stroma ratio may vary considerably and may influence protein quantitation readings significantly when an epithelial protein is concerned, e.g. a higher epithelial protein reading in tumor tissues might be solely due to the increased epithelial content of the epithelium rather than the biological overexpression of that protein. Therefore, it would be important to consider the epithelium content when we analyze the protein levels using quantitative protein assays.

There are a number of approaches to identify and quantify epithelium content from histology slides. Traditionally, the epithelium contents are read based on nuclei counts from a hematoxylin and eosin (H&E) stained histology slide by a pathologist. Another approach is to stain the histology slide with anti-cytokeratin antibody CAM 5.2 (staining for epithelia) and Masson trichrome (staining for collagenous stromal structures) [3]. More recently, with the digitization of whole slide imaging, a number of algorithms have been developed for computer-assisted readings. These methods rely on image features such as morphology, texture, color and intensity to segment images and classify them into various pathologically different regions. Automated histopathological image analysis reduces the inter-and intra-observer errors and provides additional quantitative information to aid diagnosis [4]. However, to our knowledge, these measurements have never been utilized for protein measurements.

Epithelial cell adhesion molecule (EpCAM) and cathepsin L (CTSL) are epithelial proteins that have been found abundantly expressed in prostate adenocarcinomas [5,6]. EpCAM is a well known tumor associated antigen and is expressed in various adenocarcinomas and squamous cell carcinomas (e.g. prostate, lung, colon, gastric carcinomas) [7-9]. Its expression on normal epithelia, on the other hand, is rather variable yet much lower than the carcinoma cells [10]. CTSL is a lysosomal cystein proteinase that plays a major role in the catabolism of intracellular and extracellular proteins [6]. Studies on prostate cancer cell lines suggested that CTSL was associated with the motility of prostate tumor cells and therefore might be involved in tumor metastasis [11,12]. Although previous studies suggested an increase in CTSL mRNA expression in prostate adenocarcinomas [13], a recent study showed that CTSL staining in prostate tissues is comparable between prostate adenocarcinomas and normal tissues [14].

In this study, we assessed EpCAM and CTSL levels with ELISA in prostate cancer tissues and determined the effect of epithelium content on tissue protein quantitation. To determine the effect of tissue heterogeneity on the interpretation of the ELISA result, we developed an in-house color-based segmentation method for estimation of epithelium content and applied the method on ELISA results. A pathologist estimation of the epithelium content was also applied on ELISA results. We found that both EpCAM and CTSL levels, measured by ELISA itself, were greatly affected by epithelium content in the tissue specimens. However, after normalization by epithelium percentage, ELISA measurements of both EpCAM and CTSL were in agreement with IHC staining results, demonstrating the need of normalization using epithelium content in quantitative measurement of epithelial proteins in tissue specimens.

## Materials and methods

### Materials

LSAB + Kits, biotin blocking system, antibody dilution buffer were from Dako, Carpinteria, CA. Goat-anti-CTSL antibody, antigen retrieval buffer, recombinant protein, capture and detection antibody of human CTSL, EpCAM, streptavidin-HRP conjugates and ELISA plates were from R&D Systems, Minneapolis, MN. All other chemicals were from Sigma-Aldrich (St. Louis, MO).

### Clinical specimens

Samples and clinical information were obtained with informed consent and performed with the approval of the Institutional Review Board of the Johns Hopkins University. Formalin fixed paraffin embedded (FFPE) prostate tissue slides were acquired for 6 individuals with primary prostate tumors. Additional thirty-six OCT-embedded prostate tumors were collected from radical prostatectomy at Johns Hopkins Hospital and Johns Hopkins Bayview Medical Center under the NCI-funded Johns Hopkins prostate cancer SPORE project. These tumors includes nineteen specimens with a Gleason score of 6, seven specimens with a Gleason score of 7, five specimens with a Gleason score of 8 and five specimens with a Gleason score of 9 (Additional file 1: Table S1). Eight OCT-embedded normal prostate tissues were collected from healthy transplant donors. All specimens were snap-frozen, embedded in OCT and stored at −80°C till use.

### Immunohistochemical staining and tissue microarrays

IHC staining was performed on FFPE prostate tissue slides from 6 individuals with primary prostate tumors. Sections of tissue were deparaffinized and rehydrated. Tissues were incubated in antigen retrieval buffer at 92-95°C for 10 min. CTSL was stained with Universal LSAB™ + Kits per manufacture’s protocol. Briefly, tissues were blocked by peroxidase block and 3% BSA/PBS for 30 min each followed by avidin and biotin block with Biotin Blocking System for 15 min each at room temperature. The tissues were then incubated with goat anti-CTSL primary antibody in antibody dilution buffer at 4 μg/mL followed by incubation with anti-goat biotin labeled secondary antibody and high sensitivity streptavidin-HRP for 30 min each. The CTSL staining was detected with DAB chromogen.

### Measurements of proteins from clinical specimens using ELISA

The protein samples were collected by sectioning the OCT-embedded frozen prostate tissues. The adjacent sections of about every 15 tissue sections (6 μm each) were stained with H&E for use in the computer-aided and pathologist estimation of epithelium content. For tumor specimens, the adjacent H&E slides were also used for cryostat micro-dissection to enrich the tumor tissue in the collected sample for immunoassay analysis. Places where tissues were trimmed were marked in the H&E slides and excluded for epithelium percentage estimation. An estimated number of ten to twenty 6 μm-thick tissue sections were collected in sterile screw-cap bullet tubes for each sample for protein assays. Proteins were then extracted from tissue sections using cell lysis buffer (50 mM Tris, pH 8.0, 150 mM NaCl, 0.1% SDS, 0.5% Na Deoxycholate, 1% Triton × 100). BCA assay was performed to determine and adjust the protein concentration for each tissue sample to 1 μg/mL with PBS. Tissue EpCAM and CTSL levels were then measured with ELISA assay as described before [15]. Briefly, CTSL (1 μg/mL) or EpCAM (4 μg/mL) capture antibody were coated overnight in a 96-well plate. The wells were then blocked with 3% BSA, incubated with 100 μL diluted sample, with CTSL (0.5 μg/mL) or EpCAM (0.2 μg/mL) biotinylated detection antibody for 1 h each, and with streptavidin-HRP conjugates (1:200) for 30 min. The assays were then developed with TMB substrate, stopped with H_2_SO_4_ and measured by reading the plate at 450 nm with a spectrophotometer.

### Estimation of epithelium ratio in prostate tissue specimens

An in-house color-based segmentation method was developed for estimation of epithelial areas in prostate tissue specimens using H&E stained face sections of prostate tissues. For each of the 44 cases (36 tumors and 8 normal prostate tissues), digital slides were acquired by scanning the H&E stained slide with AT Turbo (Aperio technologies, Vista, CA.). Every 2.1 × 1.3 mm^2^ area of the micrograph was then saved into a .tiff file at a resolution of 72 pixels per inch using the ImageScope software (Aperio technologies, Vista, CA.) and an estimated number of 13 ± 10 image files were generated for each one of the 44 cases. One image from each case was randomly selected to serve as the training image for the classification algorithm and the rest of the images were used as the test image set. The training images were used as the input to the classification training code. All computer simulations were implemented in MATLAB (Mathworks, Natick, MA). Each training image was segmented into four regions based on the pixel colors using a k-means clustering algorithm. K-means clustering algorithm is a clustering analysis tool for grouping a number of observations into k clusters based on the similarities between the observations. Briefly, the observations were randomly assigned to clusters for initialization and the centroid of each cluster was calculated. In an iterative manner, the cluster of each observation was updated to its nearest centroid and the centroids of the clusters were re-calculated to reflect the changes to the clusters, until the centroids converged to the optimal values. In other words, each color cluster was formed by minimizing the squared euclidean distance of the cluster members to its centroid. This will group pixels with similar colors together in a color cluster of white, bright pink, dark pink or purple in an H&E stained slide where white, pink and purple correspond to lumen, stroma and epithelium respectively. Each micrograph was arranged into 20 × 20 pixel grids such that each grid covered a 0.04 × 0.04 mm^2^ area of the tissue section. The ratio of the area of the four colors to the total area of the cell was calculated for each grid cell. Clearly, the resultant four color ratios would sum to 1 in each grid cell; thus only three of the color ratios were linearly independent. The epithelial regions of the original training image were manually marked by an experienced researcher to serve as the benchmark for the training of the classification algorithm. The marked epithelial regions are shown by a green shade. The grid cells were then divided into two groups based on whether they were marked as epithelium or not and illustrated on a scatterplot in the space of the three base colors of the H&E micrographs. Any grid cell with white content greater than 70% was marked as luminal. Knowing the class of each of the grid cells based on the marked epithelial regions, K-means clustering was again used to divide the space of the three base colors into three clusters representing the epithelial, stromal and luminal regions. These clusters, established in the space of three optionally selected color ratios, were then used to segment the images in the test dataset into epithelium, stroma and lumen area, thus estimating the percentage of each in the whole face section of prostate tissue. Segmentation code worked similar to the training code as the test image was segmented into four colors and the color ratios were calculated for each grid cell on the image. Each grid cell was classified into epithelial, stromal or luminal depending on its distance from the established clusters in the space of the three color ratios. The epithelium percentage was calculated by the following formula: Epithelium area/(Epithelium area + Stroma area) × 100%. The Matlab codes and H&E micrographs used for epithelium estimation, as well as the micrographs with estimated epithelium content are available for download at http://sdrv.ms/17tWsqd.

### Statistical analysis

Wilcoxon signed rank order test (unpaired, two-sided) was used for determination of statistical significance of EpCAM and CTSL immunoassay measurements.

## Results

### IHC staining of CTSL in prostate tissue specimens

**Figure 1:**
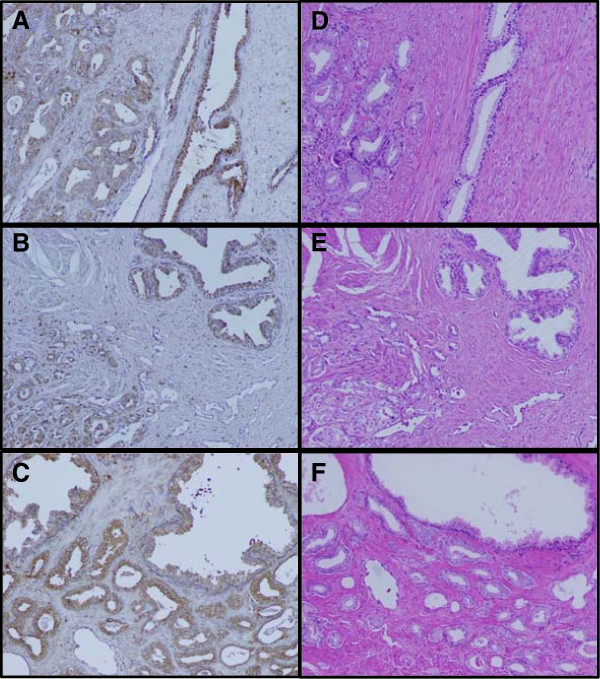
**Immunohistochemical staining of prostate tumor tissues.** Tumor tissue slides from 6 individuals were stained with anti-cathepsin L antibody and then counterstained with hematoxylin. Representative images from 3 individuals were shown in **(A)**, **(B)**, and **(C)** with corresponding hematoxylin and eosin stained reference images as shown in **(D)**, **(E)** and **(F)**.

To assess CTSL expression level in prostate tissue specimens, IHC staining was performed on 6 FFPE prostate tumor cases. Figure 1 shows representative fields of CTSL stained and H&E stained slides from 3 representative cases of the 6 FFPE cases. Similar to that reported before [14], we found that CTSL stained epithelium with no staining in the stroma compartment. Medium to strong staining of CTSL was observed in all 6 prostate tumor cases. Heterogeneity in staining intensity was observed in both tumor and adjacent normal epithelium. No differences in staining intensity were observed between tumors and the adjacent normal tissues. These results suggested that the expression of CTSL in the prostate epithelium was similar between normal and tumor tissues.

### ELISA measurements of EpCAM and CTSL in prostate tissue specimens

**Figure 2:**
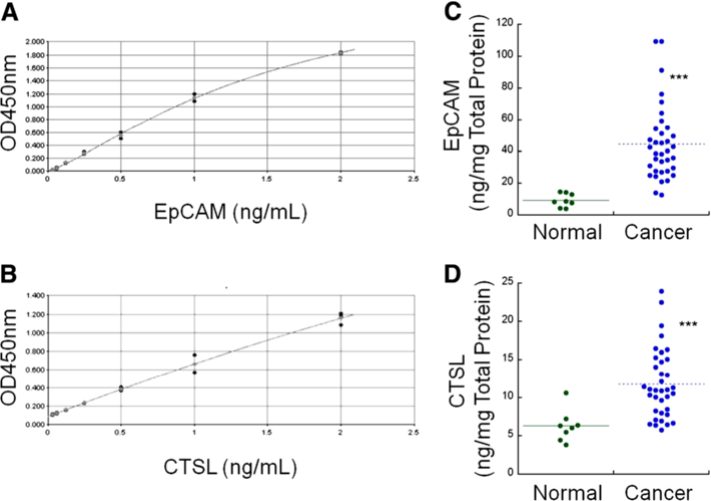
**Immunoassay measurements of epithelial cell adhesion molecule (EpCAM) and cathepsin L (CTSL) protein expression. (A)** Standard curve of EpCAM. **(B)** Standard curve of CTSL. **(C)** EpCAM measurement of tissue lysate from 8 normal prostate tissues and 36 prostate tumors. **(D)** CTSL measurement of tissue lysate from 8 normal prostate tissues and 36 prostate tumors. ***, p < 0.001, compared to normal prostate tissues.

To measure EpCAM and CTSL level in prostate tissue specimens, ELISA assays were developed for EpCAM and CTSL. The limits of detection (LOD) calculated as background OD ± 3SD were 188 and 27 pg/mL for EpCAM and CTSL respectively; the dose responsive ranges were from 0 to 2 ng/mL for both CTSL and EpCAM (Figure 2A and B). At the lowest standard point, the coefficients of variation (CV) were 0.78% and 6.3% for EpCAM and CTSL respectively. The intra-assay CVs were 3.2% and 9.9% for EpCAM and CTSL respectively. These assays were then used to analyze 8 normal prostate tissues and 36 prostate tumors. The average concentration of EpCAM was 9.39 ± 4.22 ng/mg total protein for normal tissues and 44.61 ± 23.40 ng/mg for prostate tumors (Figure 2C). The average concentration of CTSL measured was 6.30 ± 2.06 ng/mg total protein for normal tissues and 11.83 ± 4.56 ng/mg total protein for prostate tumors (Figure 2D). Compared to normal tissues, immunoassay data showed that both EpCAM and CTSL expression were significantly increased in prostate tumors with a 4.75 fold increase for EpCAM and a 1.88 fold increase for CTSL. However, as discussed earlier, since tissue protein quantitation using ELISA assay is influenced by both biological expression of the target protein and the percentage of epithelial cells expressing the proteins, further analysis needs to be done to determine the percentage of epithelial cells in order to elucidate the biological expression of EpCAM and CTSL between normal prostate tissues and prostate tumors.

### Estimation of epithelium percentage in prostate tissue

**Figure 3:**
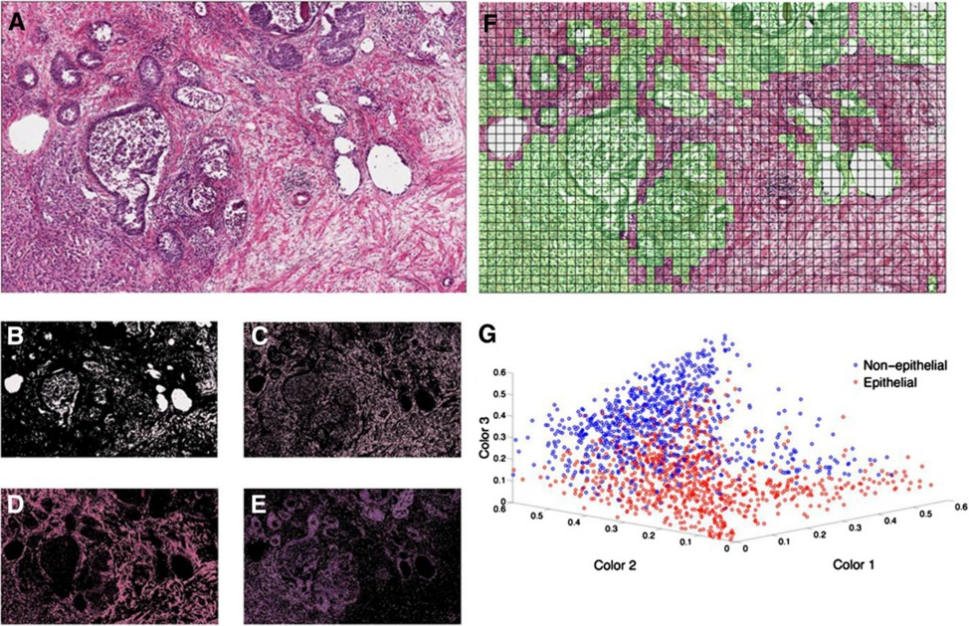
**Color based segmentation and k-means clustering of grid cells into epithelial and non-epithelial regions based on color area ratios.** A representative micrograph of prostate tissue section at 20× **(A)** is segmented into four regions based on the pixel colors of **(B)** white, **(C)** light pink, **(D)** dark pink and **(E)** purple, using a k-means clustering algorithm. **(F)** The epithelial areas of the training image were marked by an experienced prostate cancer researcher and were arranged into 20 × 20 pixel grid cells. **(G)** The four color ratios were calculated in each grid cell. Knowing the epithelial and non-epithelial regions in training sets, we classify the grid cells into two clusters. A scatter plot shows these clusters in the space of three colors, which have small overlap.

To account for the epithelium content of the tissue specimens, we used two methods: 1) estimation of epithelium content by a board certified pathologist; 2) estimation of epithelium content by a computer-aided method. The H&E stained adjacent sections of all 44 cases where CTSL and EpCAM levels were measured were analyzed for epithelium content with both methods. For the computer aided method, we developed an in-house color-based segmentation method. The 44 training H&E images, 1 from each case (Figure 3A), were segmented into four colors representing the luminal (white color, Figure 3B), stromal (light and dark pink, Figure 3C and D) and epithelial (purple, Figure 3E) regions of the tissues. Each image was then divided into grid cells each covering 0.04 × 0.04 mm^2^ of the tissue section (Figure 3F). The grid cells were already marked by a researcher as epithelial or non-epithelial. Figure 3G shows a scatter plot of the grid cells of Figure 3F in the space of the three of the four H&E base colors (white, light pink, dark pink and purple). Each dot represents one of the grid cells, which depending on its color was marked as epithelial (red) or non-epithelial (blue). The three axes of the image stand for the proportion of the pixels in each grid cell that fall into the corresponding color cluster. The non-epithelial and epithelial cloud of the dots in the defined three-dimensional space show low overlap, suggesting that a clustering algorithm can build clusters for classification of grid cells into epithelial and non-epithelial according to the color ratios in the cells. Therefore, the marked and unmarked grid cells on the training image along with their color ratios were used as inputs to the K-means clustering algorithm for generating the epithelial, stromal and luminal clusters in the space of the three color ratios. These clusters, established in the space of three optionally selected color ratios, were then used to segment the images in the test image set (13 ± 10 micrographs per case, 44 cases) into epithelium, stroma and lumen area for calculation of epithelium percentage.

**Figure 4:**
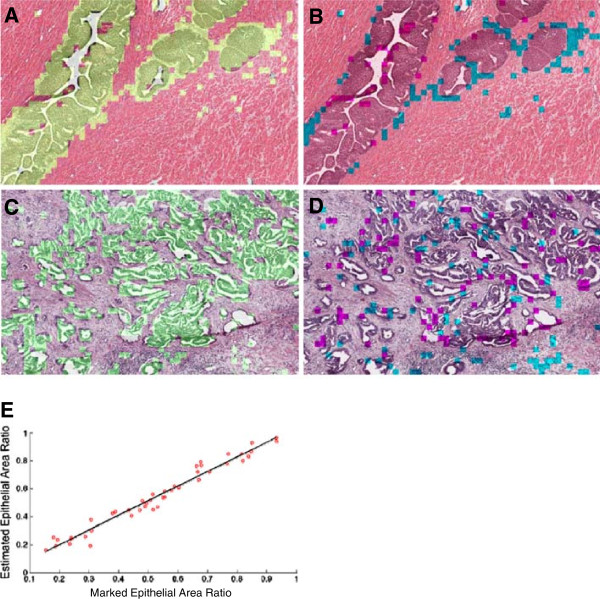
**Method output and cross-validation.** The in-house color-based segmentation algorithm was implemented on micrographs of H&E stained prostate tissue sections of 8 normal prostate tissues and 36 prostate tumors to segment the testing images into epithelial and non-epithelial regions. The method output is depicted for a normal **(A, B)** and a cancerous **(C, D)** tissue sections. In the left column, the estimated epithelial regions are highlighted in green. In the right column, the false positive and false negative regions are highlighted in blue and magenta, respectively. The set of 44 H&E histology micrographs representing 44 cases was divided into training and validation datasets. The epithelial, lumenal and stromal clusters were formed by analyzing the training set for each subject. The performance of the classifier was then evaluated by examining the training and validation dataset. **(E)** The estimated and actual epithelial areas of the validation datasets were well correlated (R^2^ = 0.965).

Figure 4A and C shows a representative micrograph of normal tissue and cancer specimen where the predicted epithelium is highlighted in green. Figure 4B and D shows the false positive and false negative regions, marked in blue and magenta respectively. Cross-validation was performed on 44 micrographs from the training image set (44 cases) to assess the accuracy of this epithelium prediction method. For this analysis, 50% of the grid cells of each of the training images were randomly selected to form the training dataset for k-means clustering, while the remaining 50% of the cells were saved to form the validation dataset for cross-validation to determine the accuracy of the method. According to cross-validation results, the method predicted epithelium ratio with an accuracy of 84.41%; with the 44 H&E histology micrographs tested, the false positive rate was 8.79 ± 5.06% and the false negative rate was 8.12 ± 5.35%. Such proximity of false positive and false negative rates improves the estimation of total epithelial area. The determination coefficient (R^2^) between the estimated epithelium area and marked epithelial area in the validation set was 0.965 (Figure 4E).

### Normalization of EpCAM and CTSL measurements with estimated epithelium percentage

**Figure 5:**
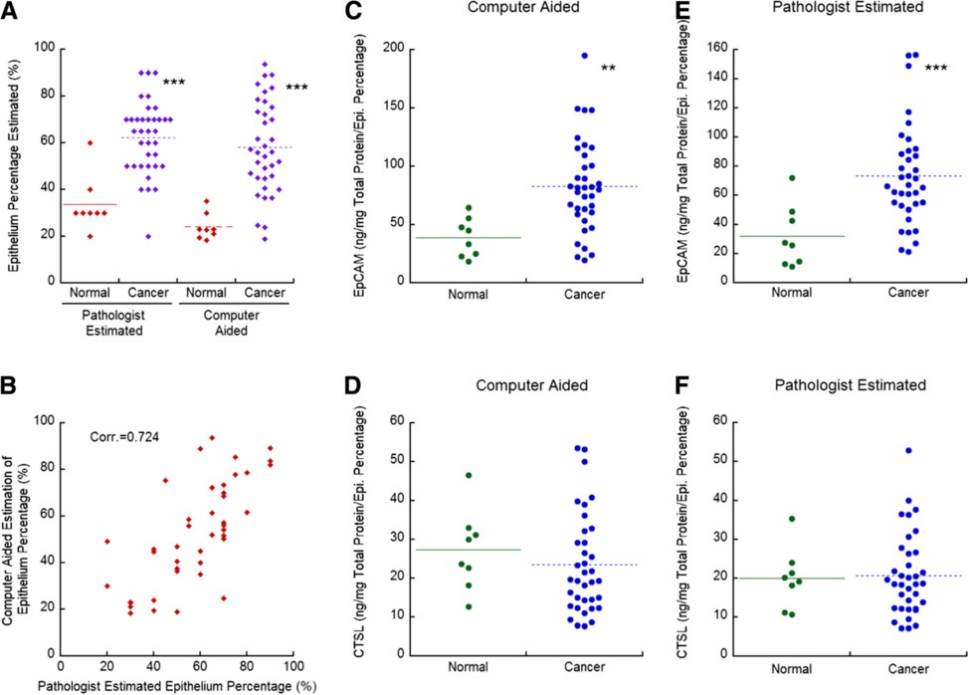
**Immunoassay measurements of epithelial cell adhesion molecule (EpCAM) and cathepsin L (CTSL) protein expression after normalization by epithelium percentage. (A)** Epithelium percentage estimated for 8 normal and 36 cancer tissue slides with computer-aided classification and pathologist estimation. **(B)** Scatter plot of computer-aided epithelium percentage estimated vs. pathologist estimation. **(C)** EpCAM ELISA measurements were adjusted with epithelium percentage estimated with the computer-aided method. **(D)** CTSL ELISA measurements were adjusted with epithelium percentage estimated with the computer aided method. **(E)** EpCAM ELISA measurements were adjusted with epithelium percentage estimated by a pathologist. **(F)** CTSL ELISA measurements were adjusted with epithelium percentage estimated by a pathologist. Corr: Pearson’s correlation coefficient. **, p < 0.01, compared to normal prostate tissue. ***, p < 0.001, compared to normal prostate tissue.

Subsequently, using the in-house developed method, we estimated the epithelium percentage of the 8 normal prostate tissues and 36 prostate tumors whose EpCAM and CTSL levels were measured. The average epithelium percentage in normal prostate tissues was 24.14 ± 5.58% (Figure 5A). This ratio was similar to what was reported before [3]. The average epithelium percentage in prostate tumors was 57.98 ± 19.75%. As expected, the epithelium percentage was significantly higher in tumors compared to that in normal tissues. Similarly, the results from pathologist estimation showed that epithelium percentage in normal prostate tissues was 33.75 ± 11.88% with 62.36 ± 15.51% estimated for prostate tumors. The computer aided and pathologist estimated epithelium percentages are statistically positively related (p < 0.001), with a Pearson correlation coefficient of 0.72 (Figure 5B).

As EpCAM and CTSL are expressed in the prostatic epithelium from IHC staining results, we used the epithelium percentage to normalize the EpCAM and CTSL ELISA results. After computer-aided normalization, the average measured EpCAM was 39.11 ± 16.69 and 82.70 ± 39.56 ng/mg total protein/epithelium percentage for normal prostate tissues and prostate tumors respectively (Figure 5C); the average measured CTSL was 27.27 ± 10.38 and 23.44 ± 12.80 ng/mg total protein/epithelium percentage for normal prostate tissues and prostate tumors (Figure 5D). After normalization by the pathologist estimated epithelium percentage, the average measured EpCAM was 32.02 ± 21.32 and 73.27 ± 34.12 ng/mg total protein/epithelium percentage for normal prostate tissues and prostate tumors respectively (Figure 5E); The average measured CTSL was 19.99 ± 7.78 and 20.74 ± 10.53 ng/mg total protein/epithelium percentage for normal prostate tissues and prostate tumors (Figure 5F). With both epithelium estimations, EpCAM expression was significantly increased in prostate tumors by about 2 fold, compared to the 4.75 fold increase without normalization (Figure 3C). In contrast to the significant elevated CTSL expression in prostate tumors from ELISA results without normalization by epithelial content (Figure 3D), CTSL expression was comparable between normal tissues and prostate tumors after epithelium normalization. These results are in agreement with previous reports where IHC staining was used to assess the protein expression of EpCAM and CTSL [10,14], demonstrating the positive impact of epithelium normalization in analyzing immunoassay results.

## Discussion

Tissue specimen is a great source for identification of disease related molecules, e.g. cancer related molecules/markers. To evaluate protein expression in tissue specimens, IHC staining is one of the most common techniques utilized. IHC staining provides insight into tissue heterogeneity, disease relevance of protein markers, and the expression pattern of protein markers in different cell types. However, IHC staining is subject to inter-observer error and is at best semi-quantitative. Direct measurements of proteins by mass spectrometry, microarray, or immunosorbent assay, on the other hand, are quantitative and can be standardized for quality assurance. Consequently, use of immunosorbent assays to measure protein expression in tissue specimens is highly desirable if the result can be properly interpreted.

In this study, we introduced epithelial percentage normalization as a tool in interpreting immunoassay results for epithelial proteins. We developed a tool for automated segmentation of micrograph slides into epithelial and non-epithelial regions using k-means clustering. K-means clustering is one the fastest and simplest clustering analysis tools that can reduce large datasets into smaller, more manageable subspaces based on the similarities observed in the dataset. The proposed epithelial percentage estimation method segments the image into four colors that are most dominant in typical H&E micrograph slides. The four colors are determined by the k-means clustering of the single pixels on each micrograph. Therefore, although these colors are categorized as white, light and dark pink and purple, they might slightly vary from one slide to the other depending on the strength of the staining on each slide. Subsequently, this method automatically accounts for the variations between the color staining on different tissue sections, which is a significant challenge in universal image processing of histology slides. In addition, this algorithm achieved an accuracy of 84% on a database of normal and prostate cancer tissue sections. The false classification of 16% was almost equally divided between the false positive and false negative results. Although the false positive and negative results are not desirable and must be minimized, in the context of epithelium percentage estimation, the inaccuracy introduced by false classifications is cancelled out to a great degree. Therefore, the equity of false positive and negative results explains the high correlation of the estimated epithelium percentage with the marked epithelial percentage, which is desirable for normalizing and interpreting the immunoassay for determining the biological changes in protein expression.

It needs to be noted that to use epithelium percentage estimation in epithelial protein measurement in tissue specimens, the H&E stain needs to be representative of the entire tissue that is studied for a given protein, and the protein needs to be measured on the same exact piece of tissue. This is because the range of epithelial to stromal ratios on a given mass of tissue varies greatly depending on the size of the tissue and the homogeneity of the tissue structures (e.g. a given prostate cancer could be 80% epithelial in some area and 5% in others). In this study, to ensure the accuracy of epithelium percentage estimated, adjacent H&E slide of approximately 15 sections of prostate tissues (6 μm each) was used for epithelium estimation.

With EpCAM and CTSL expressions in prostate tumor tissues, we demonstrated that normalization by epithelial percentage is useful in analyzing ELISA results for epithelial proteins. With IHC staining carried out in this study and in a previously published study [10,14], we showed that CTSL expression was not significantly different between normal tissue and prostate tumors after epithelium normalization. While ELISA itself misleadingly showed a significant increase of CTSL in prostate tumors, with normalization by epithelium percentage, ELISA analysis also showed that CTSL expression was comparable between these two groups. EpCAM was shown to be up-regulated in prostate carcinomas in a number of studies with IHC staining [7-9]. In this study, we also found a significant increase in EpCAM with immunoassay analysis both with and without normalization by epithelium percentage. However, the difference of EpCAM expression between tumor and normal tissues dropped significantly from 4.68 to around 2, which better depicted the biological differences of EpCAM between tumor and normal cells.

In addition to facilitate analysisof ELISA results of epithelial proteins, epithelium percentage estimation can also be used in other quantitative assays (e.g. mRNA expression, protein activity assay, clinical proteomic analysis etc.) where spatial information is lost due to sample homogenization to account for epithelium heterogeneity. With epithelium percentage normalization, improvement in the accuracy of biochemical measurements in homogenized tissue specimens may be achieved. In addition, by accounting for tissue heterogeneity, interesting new protein markers may be identified. However, further studies needs to be carried out in testing the accuracy of epithelium percentage estimation.

## Conclusions

In summary, we developed an in-house color-based segmentation method for estimation of epithelium content and demonstrated the accuracy of the method in epithelium estimation. Using EpCAM and CTSL as examples, we demonstrated that protein expressions measured by immunoassays correlate well with that measured IHC staining, suggesting that normalization by epithelium percentage is helpful in interpreting ELISA and similarly other biochemical or proteomics based assay results.

## Competing interests

The authors declare that they have no competing interests.

## Authors’ contributions

Dr. JC carried out the ELISA measurements and normalization of ELISA results by epithelium percentage and participated in the study design, manuscript writing, data analysis and development of the color segmentation based method for epithelium percentage estimation. STE developed the color segmentation based method for epithelium percentage estimation, preformed the immunohistochemical staining of cathepsin L and participated in the manuscript writing. Dr. GSB provided the clinical samples and participated in the pathologist estimation of epithelium percentages of the samples. Dr. QKL participated in the pathologist estimation of epithelium percentages of the samples. Dr. XL participated in the development of the color segmentation based method for epithelium percentage estimation. Dr. HZ participated in the study design, manuscript writing and data analysis. All authors read and approved the final manuscript.

## Supplementary Material

Additional file 1: Table S1 Clinical information of normal prostate and prostate tumor tissues employed in this study. Click here for file
